# Correlation Analysis Study Between the Intensity of Counseling Friendship Network and Exercise Time Among University Taekwondo Athletics

**DOI:** 10.1002/brb3.70264

**Published:** 2025-06-10

**Authors:** Sung‐Min Son, Byung‐O Ahn

**Affiliations:** ^1^ Future‐Academic Cooperation Foundation Yonsei University Wonju‐si Ganwon‐do South Korea; ^2^ Law Firm Hambaek Seoul‐si South Korea

**Keywords:** correlation analysis, counseling friendship network, eigenvector centrality, exercise time, social network analysis, university taekwondo athletics

## Abstract

**Background:**

The counseling friendship is defined as a relationship based on affection and mutual trust, wherein individuals share care, interest, and information, such as exchange emotions and concerns, thereby functioning as a social support system. Analyzing counseling friendships among university taekwondo athletes provides essential foundational data for overcoming the challenges they face and enhancing their athletic performance.

**Purpose:**

The purpose of this study is to analyze the correlation between the intensity of counseling friendship networks and exercise time among university taekwondo athletes by using social network analysis (SNA).

**Methods:**

The study subjects were 69 university taekwondo athletes. To analyze the intensity of the counseling friendship network, SNA was employed, focusing on the indices of network distribution and centrality. The counseling friendship assessment was conducted using a structured friendship survey, allowing athletes to list the names of up to three friends from whom they sought advice related to their university and athletic lives. The strength of these friendships was quantified based on the duration of their acquaintance. Additionally, measurement of exercise duration was conducted using a 4‐point scale to assess the average daily exercise time, with intervals set for up to 4 hour or more. The analysis aimed to explore both the network distribution index and the centrality index within the context of these counseling friendships.

**Results:**

As a result, a positive correlation was shown between eigenvector centrality and exercise time, and as the level of eigenvector centrality increases, the exercise time increases.

**Conclusions:**

Based on this, to improve exercise time, it is necessary to create a way to support counseling through friendships and establish a program that allows counseling relationships to be formed centering on friends with a high level of centrality.

## Graphical abstract

Counseling friendships provide psychological stability for university taekwondo athletes, which in turn aids in enhancing their athletic performance. Athletes with stronger counseling friendships are more likely to experience increased exercise duration. To increase exercise duration, it is beneficial to form relationships with friends who offer effective advice and support; particularly, relationships with friends who hold key positions within the counseling network are more effective for extending exercise time.

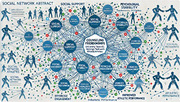

## 1 Introduction

Friendship defines a companionable relationship where individuals maintain intimacy through voluntary meaningful interactions, sharing affection, interest, and information, forming a dyadic attachment based on affection and trust (Sharabany, Gerhoni, and Hofman 1981). K. Yu and Yi (2015) conceptualized friendship in their study as a relationship characterized by frequent and intense mutual interactions. Additionally, (M. Lee, Shin, and Kang 2018) defined friendship as a reciprocal and meaningful connection involving mutual understanding, sharing personal thoughts and emotions, and even divulging secrets.

In this regard, friendship serves a crucial function in acquiring fundamental elements within social relationships. It enables individuals to evaluate and reflect upon themselves through the actions of others, fostering social growth (Sharabany, Gerhoni, and Hofman 1981). From a psychosocial perspective, friendships induce psychological stability, help overcome various conflicts and value discrepancies, and allow the formation of one's self‐concept, thereby promoting psychosocial development. Consequently, for university students preparing for personal growth as social beings, friendships play a vital role, serving functions that facilitate social development and maturity (Song and Lee 2011).

Friendships are characterized by a foundation based on specific purposes, involving the joint undertaking of purpose‐based activities to maintain and foster the relationship. Consequently, the objectives of a relationship directly influence attitudes, behaviors, and social development within the context of friendships (You 2007). Among various friendships with specific purposes, counseling friendships are defined as relationships to address and resolve various concerns and problems encountered in daily life. These relationships play a crucial role as social support systems, involving the sharing of emotions and feelings related to concerns and issues, as well as the exchange of information (Song and Lee 2011).

Recent studies have reported that counseling with friends positively influences athletic performance and exercise immersion (MacGeorge et al. 2016). According to these studies, individuals who receive advice can gain valuable information and insights, experience a reduction in pain, be persuaded to take advising actions, and develop positive feelings toward those providing advice (Arora et al. 2007). Additionally, Ambroży et al. (2024) emphasized the importance of focusing on beliefs that enhance one's skills, acquire knowledge, strive for success, seek to understand tasks, and collaborate with peers when defining task‐oriented goals, such as athletic achievement and performance. In this context, O'Keefe, O'Keefe, and Lavie (2018) argued that social interactions influence an individual's exercise participation and psychological stability, contributing to a reduction in life expectancy and cardiovascular risks. Indeed, Dharia et al. (2016) and Fitzgerald, Fitzgerald, and Aherne (2012) highlighted the significant importance of interactions with friends related to exercise duration and performance, asserting that such interactions play a critical role in achieving exercise goals. These studies suggest that counseling and the quality of friendships contribute to individual exercise immersion and performance, thereby further underscoring the necessity of the current research.

Exercise duration serves as a crucial indicator in assessing the level of exercise immersion and plays a pivotal role in enhancing the effectiveness of physical activity (Kim and Kim 2007). Specifically, exercise duration contributes to strengthening muscular and cardiovascular endurance, providing sustained stimuli that induce both physical and mental transformations (I. Park and Kim 2013). Consequently, allocating a certain amount of time to exercise becomes imperative in managing and promoting individual health in everyday life (Kim and Kim 2007). For athletes, exercise duration holds significant importance, playing a key role in improving athletic performance and achievement (S. Lee 2006).

University taekwondo athletes refer to individuals who are affiliated with a university as taekwondo players, balancing their academic pursuits, university life, and life as taekwondo practitioners (J. Yu, Lim, and Bae 2021). These athletes face various challenges in their daily lives as they strive to achieve their desired goals (Yun, Kim, and Seo 2020). Particularly, they encounter numerous obstacles and limitations throughout their university life, academic endeavors, and overall athletic journey. Consequently, based on their characteristics and the extent of the challenges they face, some athletes may opt to discontinue their athletic pursuits if the difficulties become severe. Additionally, they often experience high levels of stress and psychological anxiety (J. Yu, Lim, and Bae 2021).

Accordingly, research focused on university‐affiliated taekwondo athletes provides a crucial foundation for overcoming the challenges and issues they face, offering essential insights to address and resolve these issues. By offering appropriate alternatives, this research becomes a valuable resource for taekwondo athletes to achieve high levels of success in their athletic pursuits. Furthermore, this study can serve as significant evidence in establishing strategies to address the various problems and challenges faced by taekwondo athletes, contributing to the formation of a foundation that enhances sports engagement and athletic performance.

Research on university taekwondo athletes has been extensively reported, primarily focusing on psychosocial variables. However, studies that set their friendships and exercise duration as variables are notably scarce and limited. Furthermore, despite the recognized impact of friendships on the resilience, exercise immersion, and athletic performance of university taekwondo athletes, research specifically examining friendships as a variable is still lacking. Therefore, the objective of this study is to utilize social network analysis (SNA) to analyze the relationship between the intensity of counseling friendships among university taekwondo athletes and their exercise duration. This research aims to address the existing gap in the literature by exploring how the strength of counseling friendships is related to the amount of time spent on physical activity among university taekwondo athletes.

Research Questions

1. What are the characteristics and strengths of the counseling friendship relationships among university taekwondo athletes?
2. Is there a correlation between the strength of counseling friendship relationships and exercise time among university taekwondo athletes?

Research Hypothesis

1. The strength of counseling friendships among university taekwondo athletes will be related to exercise time.

## 2 Methods

### 2.1 Study Subjects

This study targets taekwondo athletes affiliated with University I in Gyeongsangnam‐do Province, South Korea. The criteria for selecting participants are as follows: (1) university students affiliated with the taekwondo team and (2) individuals who understand the purpose and content of the study and voluntarily agree to participate. Exclusion criteria for the study include individuals taking psychotropic drugs that may induce behavioral issues during the evaluation process.

The consent process for participation in this study followed the principles of the Helsinki Declaration and proceeded as follows: potential participants were provided with an explanation of the purpose and content of the study, ensuring their full comprehension. Subsequently, they were allowed to voluntarily consent to participation. Individual consent for the study was obtained in writing.

### 2.2 Study Procedures

This study was conducted using a single‐group quasi‐experimental research design. First, the methodology of SNA was employed to analyze the strength of counseling friendships among the study participants. Additionally, correlation analysis was utilized to examine the relationship between the strength of counseling friendships and exercise duration. The data collection for each variable in this study was conducted individually by the primary researcher in a lecture room at University I. To minimize environmental distractions that could affect participants' concentration, the surroundings were organized before each variable assessment. Before the assessments, a comprehensive explanation of the study's purpose and methodology was provided. The study was conducted over three months, from March 1, 2023, to June 30, 2023

### 2.3 Study Tools

#### 2.3.1 Analysis of Counseling Friendship Network Intensity

In this study, we adopted the method of SNA, a social science analytical approach, to analyze counseling friendship network intensity. To investigate these relationships, a structured questionnaire was employed. The questionnaire focused on the friendships through which university‐affiliated taekwondo athletes seek advice in their university and athletic lives. Participants were free to provide the names of up to three friends, along with details such as each friend's gender and the frequency of counseling sessions within a week. We established a duration parameter to assess the intimacy of counseling friendships, allowing respondents to specify this period in months. Based on this information, we analyzed counseling friendships. Additionally, participants could indicate “None” or leave the space blank if they did not have friends corresponding to the criteria.

#### 2.3.2 Exercise Time

In this study, the analysis of exercise duration was conducted based on Bae's research (Bae 2017), using a 4‐point scale. The scale ranged from 1 point for 1 h or more but less than 2 h/day, 2 points for 2 h or more but less than 3 h/day, 3 points for 3 h or more but less than 4 h/day, to 4 points for 4 h or more per day. A higher score indicates a higher amount of time dedicated to exercise.

### 2.4 Statistical Analysis

The collected data for each item were encoded and analyzed using specific software according to the respective methodologies. For SNA, Netminer 4.0 software (Cyram, Co. Korea) was utilized. The analysis of the general characteristics of the participants and the impact of counseling friendships on exercise duration was conducted using IBM SPSS Statistics 26. Descriptive statistics were employed for the analysis of participants' general characteristics. The relationship between counseling friendship intensity and exercise duration was examined using correlation analysis. In this study, a significance level of *p* < 0.05 was applied.

Before conducting the statistical analysis, the internal consistency of each variable was analyzed. The results for the counseling friendship network intensity showed a Cronbach's alpha value of 0.94, while the results for exercise time showed a Cronbach's alpha value of 0.92.

## 3 Results

### 3.1 Characteristics of Study Subjects

The analysis of the participants' general characteristics is presented in Table 1. The study included a total of 69 university taekwondo athletes. Among the participants, 54 were male (78.26%), and 15 were female (21.74%). Regarding the academic year, there were 9 participants in the first year (14.06%), 14 in the second year (20.29%), 22 in the third year (31.88%), and 24 in the fourth year (34.78%). The total years of athletic experience were distributed as follows: less than 3 years (15 participants or 21.74%), 3–5 years (29 participants or 42.03%), 6–9 years (18 participants or 26.09%), and 10 years or more (7 participants or 10.14%).

**TABLE 1:** Characteristics of study subjects (*n* = 69).

Sex (*n*, %)		Grade (*n*, %)		Total athletic’ careers (*n*, %)	
Male	54 (78.26)	1 grade	9 (14.06)	3 years below	15 (21.74)
		2 grades	14 (20.29)	3–5 years	29 (42.03)
Female	15 (21.74)	3 grades	22 (31.88)	6–9 years	18 (26.09)
		4 grades	24 (34.78)	over 10 years	7 (10.14)

### 3.2 Analysis of Network Distributions

The results of the network distribution analysis are presented in Table 2. The density was found to be 0.035, indicating cohesion of 3.5%. The average degree centrality was 2.348. The inclusivity (completeness) was 1, indicating the inclusion of 100% of all nodes, and thus, no isolated nodes were observed.

**TABLE 2:** Results of network characteristics.

Indicators	Density	Mean degree	Inclusiveness	Isolation (*n*)
Values	0.035	8.40	1.00 (100%)	0

### 3.3 Results of Network Centrality

**FIGURE 1:**
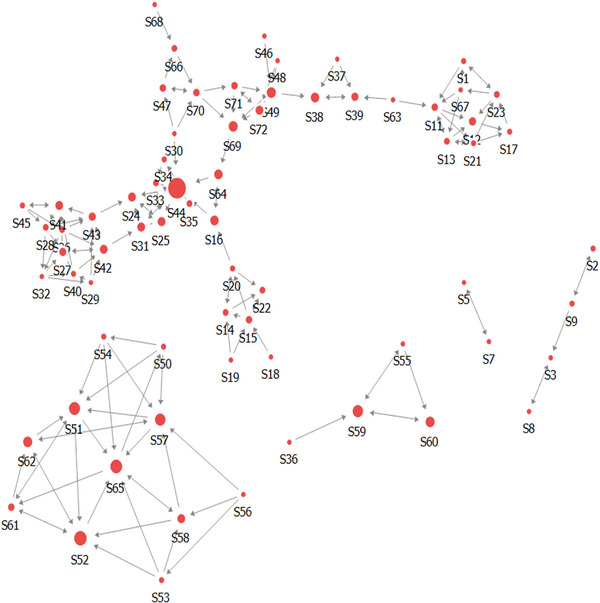
Counseling network structure.

The network centrality was analyzed by distinguishing between degree centrality and betweenness centrality, taking into consideration the directionality as in‐degree (in) and out‐degree (out). The results are presented in Table 3 and Figure 1. The average in‐degree centrality was 1.325, with a minimum of 0 and a maximum of 7.206. The average out‐degree centrality was also 1.325, with a minimum of 0.103 and a maximum of 4.059. Notably, the maximum value of average in‐degree centrality was higher, indicating a relatively higher proportion compared to the average out‐degree centrality.∖

**TABLE 3:** The results of network centrality.

Indicators	Mean	S.D.	Min	Max
In‐degree centrality (values)	1.325	1.294	0	7.206
Out‐degree centrality (values)	1.325	0.837	0.103	4.059
Betweenness centrality (values)	0.032	0.06	0	0.244
Eigenvector centrality (values)	0.043	0.113	0	0.581

The average betweenness centrality was 0.032, ranging from a minimum of 0 to a maximum of 0.244. The average eigenvector centrality was 0.043, with a minimum of 0 and a maximum of 0.581. The average eigenvector centrality was relatively higher than the average betweenness centrality. These results suggest that, based on the significance of the indicators within the network, there is a tendency for relationships to be centered around individuals with a high degree of centrality rather than forming mutually connected relationships.

### 3.4 Results of Correlation Between Counseling Network Intensity and Exercise Time

The results of the correlation analysis are presented in Table 4. A significant positive correlation (*r* = 0.292) was observed between eigenvector centrality and exercise duration at a 95% confidence level. Additionally, eigenvector centrality showed significant positive correlations with in‐degree centrality (*r* = 0.511), out‐degree centrality (*r* = 0.489), and betweenness centrality (*r* = 0.574) at a 99% confidence level. These findings suggest that as in‐degree centrality, out‐degree centrality, and betweenness centrality increase, the level of eigenvector centrality also increases. Moreover, higher levels of eigenvector centrality are associated with increased exercise duration.

**TABLE 4:** Results of correlation analysis.

Centrality	In‐degree centrality	Out‐degree centrality	Betweenness centrality	Eigenvector centrality	Exercise time
In‐degree centrality	1	0.492^**^	0.460^**^	0.511^**^	0.154
Out‐degree centrality	0.492^**^	1	0.133	0.489^**^	0.181
Betweenness centrality	0.460^**^	0.133	1	0.574^**^	0.078
Eigenvector centrality	0.511^**^	0.489^**^	0.574^**^	1	0.292^*^
Exercise time	0.154	0.181	0.078	0.292^*^	1

* *p* < 0.05.

** *p* < 0.01.

## 4 Discussion

This study utilized SNA to analyze the strength of counseling friendships among university taekwondo athletes and their relationship with exercise duration. SNA is a methodology that focuses on analyzing relationships among individuals, particularly in the context of friendships. By examining the structural characteristics of friendship networks, SNA allows for the understanding of the features of these relationships and provides insights into the behaviors that emerge from such friendships, facilitating practical applications in real life (Choi and Jung 2009). Furthermore, SNA enables the analysis of both individual and group‐centered perspectives, allowing for an understanding of how individual behaviors influence the group and vice versa. It helps identify an individual's position within the group, the roles they play, and the extent of their influence within the group (M. Lee, Shin, and Kang 2018).

Quantitative research employs methodologies centered around individuals, utilizing quantitative indicators such as the number of friends to describe and generalize the characteristics of friendships (M. Lee, Shin, and Kang 2018). However, these studies have limitations as they do not consider the mutual nature of friendship relationships and are restricted in exploring the structure of friendships based on real‐life contexts (S. Park and Hong 2008). On the other hand, qualitative research focuses on understanding the functional aspects of friendships, allowing for the exploration of various abstract elements within these relationships. However, qualitative research lacks the statistical verification process for the analyzed results, leading to limitations in ensuring reliability and validity. Additionally, it fails to capture the structure of relationships and the resulting behaviors (Kang et al. 2020).

This study utilized SNA to present the structural characteristics of counseling friendships among university taekwondo athletes. It not only illustrated the individual's position within the network but also structurally depicted the characteristics of these friendships on a collective level, including their influence. Through this analysis, the study provided insights into the features of friendship relationships that need consideration for enhancing the exercise time of taekwondo athletes. Therefore, by employing SNA, this study addressed the limitations of quantitative and qualitative research commonly used in friendship analysis, providing a comprehensive understanding. The results of this study are deemed academically meaningful and valuable, offering foundational information for the development of specific strategies and approach to enhance exercise time for university taekwondo athletes.

The centrality metrics in SNA describe the extent to which an individual is positioned within the network of counseling friendships, indicating the importance of their role in these relationships. These metrics help assess whether an individual plays a significant role in the structural aspects of counseling friendships, allowing for an evaluation of their influence within the network (Choi and Jung 2009). Among these metrics, closeness centrality is particularly valuable, reflecting the importance and connectivity of other friends in the network. High closeness centrality suggests a friend has considerable influence in counseling friendships, indicating a substantial impact on the overall network (Kho, Cho, and Cho 2013). Therefore, based on these findings, the results of this study suggest that as individuals form relationships with friends who excel in counseling and providing advice, there is an associated increase in the amount of time devoted to physical activities.

Results of the analysis on the relationship between friendship dynamics and exercise duration provide valuable insights for understanding the structural characteristics of counseling friendships among university taekwondo athletes. These findings go beyond individual‐level considerations, offering a broader perspective on the nature of these friendships and their collective influence.

Counseling provides university taekwondo athletes with a platform to share and discuss various concerns and challenges they face in their university life, athletic endeavors, and daily routines (Song and Lee 2011). Through these discussions and shared experiences, counseling facilitates problem‐solving and enables individuals to focus more on their athletic pursuits. Based on the results of this study, it is deemed important to devise strategies to strengthen counseling and support within the friendships of university‐affiliated taekwondo athletes. This may involve creating programs that encourage the formation of relationships centered around friends who excel in providing counseling and advice. The implementation of various strategies will be crucial for addressing the diverse needs identified in this study.

## 5 Research Limitation and Future Directions

This study has several limitations. First, it focused on a single group of participants, limiting the generalizability of the results to university‐affiliated taekwondo athletes only. Subsequent research should consider conducting comparative analyses with various groups to enhance the applicability of the findings. Additionally, the influence of friendship on exercise time can vary widely. Therefore, it is crucial to compare and analyze the relationship between exercise time and various types of friendships formed by university taekwondo athletes. Understanding which types of friendships impact exercise time can be valuable for incorporating into training processes.

Furthermore, this study solely analyzed the correlation between counseling friendships and exercise time among university taekwondo athletes. As a result, it did not establish a causal relationship between counseling friendships and exercise time. Future research should build upon these findings to explore the causal link between counseling friendships and exercise time.

Looking forward, future research endeavors should consider delving into more nuanced investigations. Exploring diverse groups and conducting comparative analyses could provide a broader understanding of the impact of counseling friendships on exercise time. Additionally, investigating causation in the relationship between counseling friendships and exercise time would contribute to a more comprehensive comprehension of these dynamics. Further studies could explore the specific mechanisms through which counseling friendships exert influence, leading to the development of targeted interventions for optimizing exercise behavior among university taekwondo athletes.

## 6 Conclusions

This study has successfully employed SNA to scrutinize the strength of counseling friendships among university taekwondo athletes and has explored its association with exercise time. The results underscore a significant positive correlation between exercise time and the strength of counseling friendships, particularly concerning eigenvector centrality. Notably, a higher level of eigenvector centrality is linked to an increase in exercise time.

These findings offer pivotal insights for formulating strategies aimed at improving exercise time among university taekwondo athletes. Building upon these results, it is advisable to develop interventions that promote counseling through friendships, thereby enhancing exercise engagement. Moreover, implementing programs designed to foster the establishment of counseling relationships, especially with friends exhibiting high centrality levels, holds promise for positive outcomes.

## Author Contributions

**Sung‐Min Son**: investigation, funding acquisition, writing–original draft, validation, visualization, writing–review and editing, formal analysis, supervision, resources, project administration. **Byung‐O Ahn**: conceptualization, investigation, funding acquisition, writing–original draft, methodology, visualization, writing–review and editing, validation, software, formal analysis, project administration, data curation, resources, supervision.

## Ethics Statement

The research received approval from the Kangwon National University Institutional Review Board (KWNUIRB‐2021‐06‐003‐003).

## Consent

The authors have nothing to report.

## Conflicts of Interest

The authors declare no conflicts of interest.

## Peer Review

The peer review history for this article is available at https://publons.com/publon/10.1002/brb3.70264.

## Data Availability

The data that support the findings of this study are available from the corresponding author upon reasonable request.
